# What? When? And How?

**Published:** 1890-10-18

**Authors:** Jonathan Hutchinson

**Affiliations:** ex-President of the Royal College of Surgeons; Consulting Surgeon of the London Hospital.


### INTRODUCTORY LECTURE.

### WHAT? WHEN? AND HOW?

By Jonathan Hutchinson, LL.D., F.R.S., ex-President of the Royal College of Surgeons; Consulting Surgeon of the London Hospital. Being the substance of an address delivered at the opening meeting of the University College Medical Society on October 8th.

In commencing, he said: The subject of my remarks is education, and especially the branch of education that a medical man has to go through. Education, may be defined as the accumulation of experience, or the sum of that which fits men to make good use of the experience of life. A good educated man is one who profits by his experience. This being our definition of education, the next question is what should be the kind of education of most use to a medical student. That is to say, What should he get? When should he get it? and How should he get it? These are the problems we have to deal with.

At the present day in some quarters there is a desire to minimise the preliminary education. It is, however, a great question whether we do wisely to cut out some of the preliminary subjects.

I believe that the student, no more than the examiner, desires to exempt himself from subjects which might be of use to him in after life. Let us first look at those subjects which some consider should be made optional. I maintain that the education of a medical man should be the same as is considered part of the education of a gentleman. In the first place Greek, I consider as most desirable, so many words used in the various branches of our science are derived from the Greek, and it follows that anyone not knowing these derivations must be at a disadvantage compared with those who have this knowledge. I am not one who would urge men to spend time on subjects which are not of commensurate value to them, but a knowledge of the Greek vocabulary is essential. Some have said that John Hunter was very badly educated in the preliminary branches of his profession, and yet he was a most wonderful man, but to this I would reply that Hunter was a genius, but how much better still might he have done if his preliminary education had been more complete?

Now passing to Latin, my opinion is the same. If we know what our subject is, we shall know how to proceed. We do not want to write Latin verses, but we do want to know the Latin derivation of words; this is enough, a detailed and accurate knowledge is not essential. How, then, should they be learnt? We should not begin by learning Latin grammar, but we should begin by vocabularies. Let us, then, next read freely with the help of translations. It is quite possible to get enough knowledge of Latin without the grammar. Latin and Greek are the two implements for our future work, we learn the use of them, we do not want to pursue them further.

Now as regards French and German. In these modern times we all travel, and so all need these languages; so also a vast amount of scientific literature is written in both of them. But for all this, I cannot say that they are as essential as Latin and Greek. Here we must make a suggestion as to the How! If anyone wants to learn one of these languages he must go where they are spoken, and not labour with the grammar at home. Now we pass on to familiarity with general literature. Many of us in early life have acquired tastes for literature. Let us, then, not drop these. You should read novels, history, and general literature. Literature intensifies our feelings, and these feelings are the basis upon which we form our ideas on general subjects. The object of all culture is to bring us into sympathy with others. As the basis of physiology and anatomy are essential to medical knowledge, so the study of literature forms a basis of our dealings one with the other.

Geography, even looking at this subject from a purely medical point of view, a knowledge of it is necessary, and should be acquired in early life. Again, a word as to how! In my younger days this subject was very largely taught from globes. Now we learn it from lists of names of rivers, towns, &c., from books. It should be learnt objectively—that is, by making maps ourselves.

History again becomes of great importance with reference to the development of different diseases in different ages, in additon to its importance as a branch of general literature.

Concerning algebra and mathematics I will not trouble you, but they may be considered as luxuries, and not essential to a medical man.

Zoology—this subject all will agree with me is of the greatest importance to the student of man, not only with regard to the normal anatomy and physiology of the lower forms of animals, but also with regard to the pathological states, and I should recommend a course of study applied to the diseases occurring in lower animals as a preliminary to the study of disease in man.

Botany, also, is of great importance, and I think that a medical man should know and be able to recognise the common plants when he sees them in the field. I well remember when a boy going round the garden one day with our family doctor, a man for whom I had the greatest respect. We showed him some very fine specimens of Belladonna lilies. He exclaimed, "I am surprised that your mother should keep these poisonous plants in the garden with so many children about." He even thought that these lilies were the Atropa Belladonna. From that moment I lost all respect for that man. Another with whom I was travelling mistook a large group of equisetums for young fir trees. I would study botany by going straight to the plants themselves, pick the flowers, and then yourself dissect them. A grasp of what we might call herbalistic botany is essential, a grasp of plant life is of great interest. The knowledge of the growth of cryptogams is of great importance in relation to many forms of skin disease, many of these being due to the growth of these plants in the skin.

So again, Geology—one wishing to learn something of this should go to the objects and not first to books; one will then soon find that there is nothing mysterious about the subject, but all is plain. A medical man should know something of Meteorology—I do not mean to say that he should be able to prognosticate the weather, but he should know, for instance, whether the dew falls or rises, he should know if the dew comes from above or from below. For the subject of Chemistry I have no time to speak, but must pass on to Human Anatomy and Physiology. These, again, should be learnt in a practical and not a pedantic manner. It would be well if more attention were paid to fundamental subjects What is essential to the professor is not necessarily essential to the practical man of medicine.

Some people have held the most extraordinary ideas concerning the education of classes of medical men. I will read you a few lines written by a Professor Ryle, who wrote about a century ago: "Upon the education of medical men for the country folk, called 'routiners,'" after saying that they should have a smattering of several subjects, such as toxicology, pharmacy, anatomy, medicine, etc., writes, "and he must exactly resemble the common people among whom he lives both in manner of dress, habits, and method of thought." After all, the difficulties of education lie in what we call memory. So much we very soon forget, but I am often disposed to think that, in reality, what we forget we never knew. We should endeavour to learn subjects in such a manner as not to tire or fatigue the memory. I know no more about what is called the physical basis of memory than that it has to do with the cells of the grey matter of the brain, and these must be kept in good nutritive condition or they will not work well. From this it follows that we, as medical men, should insist upon the importance of all schools being in healthy places, in order to develop to the highest point those receptive powers which are essential to the well-being of the student. The advantages of a student's life being spent in healthy and invigorating localities is all important. With regard to artificial aids to memory I have but little faith in any of these, when they are used they should be of the simplest possible kind. But with regard to this point we should as much as possible associate, especially cultivating methods of natural associations. I will give you an instance. The battle of Issis was fought in the year 33 B.C., there being three s's in Issis. This battle was fought by Alexander the Great. With these few facts what a host of associated ideas at once come up to the mind.

As I have already laid so much stress upon objective learning, I will speak further upon it. Objective studies are easily retained; studies from books are soon forgotten. We must avoid, so to speak, throwing so many books upon our brains that the brain cannot move. So also you should associate studies with pleasure, or rather I would say do not dissociate work and holidays.

If we have a true love for our profession we shall never care to throw up all interest in it for some weeks every summer, or at other periods. With regard to the objective study, of which I have already said so much, I would lay stress upon study in museums; that is to say, in good museums; I would also lay great stress on the value of photographs and models; these are all far superior to books for the purpose of study. In medicine and surgery go into the wards and yourself see the cases from day to day, and do not trust for your source of information too much to books. To my mind drawing is of all importance to a student; let him learn to draw, even if only for the purpose of diagrams.

Let him also learn to cultivate the art of conversation, and with this the teaching of others. If you wish to remember a good joke you tell it to some one else, and the same you should do in your daily work. In addressing a society such as this one, in which we teach each other, these words will be understood. In teaching we learn, giving we receive. When a man ceases to teach he ceases to learn, and conversely, I maintain, that when a man ceases to learn he should also cease to teach. If we wish to learn we should engage in teaching. With regard to the student, I think he should have more liberty to go where he wishes to learn, and not be forced to attend any one teacher or course of lectures. The student knows himself where he can learn far better than we can dictate to him. The medical profession now stands at the head of the learned professions; there was a day when it was not so, and when the clerical profession took the lead; times have rapidly changed, and this is no longer so. I conclude with the words, "The difference between an ignorant man and a learned man, is the difference between a man who is alive and a man who is dead."

